# Peripapillary Hyperreflective Ovoid Mass‐Like Structure as an Optic Disc Pseudo‐Edema Case Report and Review

**DOI:** 10.1155/crop/9417297

**Published:** 2026-02-09

**Authors:** Rafaela Saalfeld, Ana H. Hirata Choi, Leonardo P. Zeni, Manuel A. P. Vilela

**Affiliations:** ^1^ Retina Department, Post-Graduation Program, Ivo Corrêa-Meyer Institute, Porto Alegre, Brazil; ^2^ Clinical Surgical Department, Federal University of Health Sciences of Porto Alegre, Porto Alegre, Brazil, ufcspa.edu.br

**Keywords:** optic disc drusen, optic disc edema, optic nerve, optical coherence tomography, peripapillary hyperreflective ovoid mass-like structures

## Abstract

**Background:**

The peripapillary hyperreflective ovoid mass‐like structure (PHOMS) has been defined by the Optic Disc Drusen Studies (ODDS) Consortium as a distinct finding separate from the optic disc drusen (ODD). Given that PHOMS is neither a precursor nor subtype of ODD, its role as an independent risk factor for optic nerve diseases and its secondary consequences on nerve fibre function remain unclear.

**Case Presentation:**

We present the case of a 12‐year‐old child diagnosed with PHOMS. Multimodal ophthalmic imaging, including optical coherence tomography (OCT), revealed a peripapillary hyperreflective ovoid mass without calcification and elevated disc margins, but no signs of increased intracranial pressure or subretinal fluid, thereby distinguishing PHOMS from papilledema. Visual acuity was preserved, and no neurological abnormalities were detected.

**Conclusions:**

This case illustrates important clinical and imaging distinctions between PHOMS and papilledema, emphasising the need for dedicated examination of PHOMS across disease groups. Longitudinal studies involving larger cohorts are warranted to determine whether PHOMS can serve as a biomarker for the magnitude and severity of optic nerve damage.

## 1. Introduction

In 2015, The Optic Disc Drusen Studies (ODDS) Consortium, while seeking to establish a consensus recommendation for the diagnosis of optic disc drusen (ODD) using optical coherence tomography (OCT), categorized a peripapillary hyperreflective ovoid mass‐like structure (PHOMS) as a distinct entity separate from ODD [1]. PHOMS is linked with herniations and torsions of optic nerve fibres stemming from axoplasmic stasis and congestion in the lymphatic transluminal pressure system [2, 3]. These findings are now observed with a wide prevalence range in seemingly normal eyes and various ocular and neurological conditions, including papilledema, ODD, nonarteritic anterior ischemic optic neuropathy (NA‐AION), optic neuritis, neuromyelitis, multiple sclerosis, Leber hereditary optic neuropathy, myopia, melanocytoma and tilted discs, all of which share a common pathophysiological process of axonal stasis and herniation [4–8].

PHOMS can be classified into three categories: (1) optic disc edema associated, (2) ODD associated, and (3) anomalous disc associated. Although PHOMS is considered a biomarker of axoplasmic stasis, its distinction from ODD—as neither a precursor nor a subtype—leaves uncertain whether it represents an independent risk factor for optic nerve disease or affects nerve fibre function. We report the case of a 12‐year‐old child with PHOMS and highlight its key differences from optic disc edema.

## 2. Case Report

**Figure 1:**
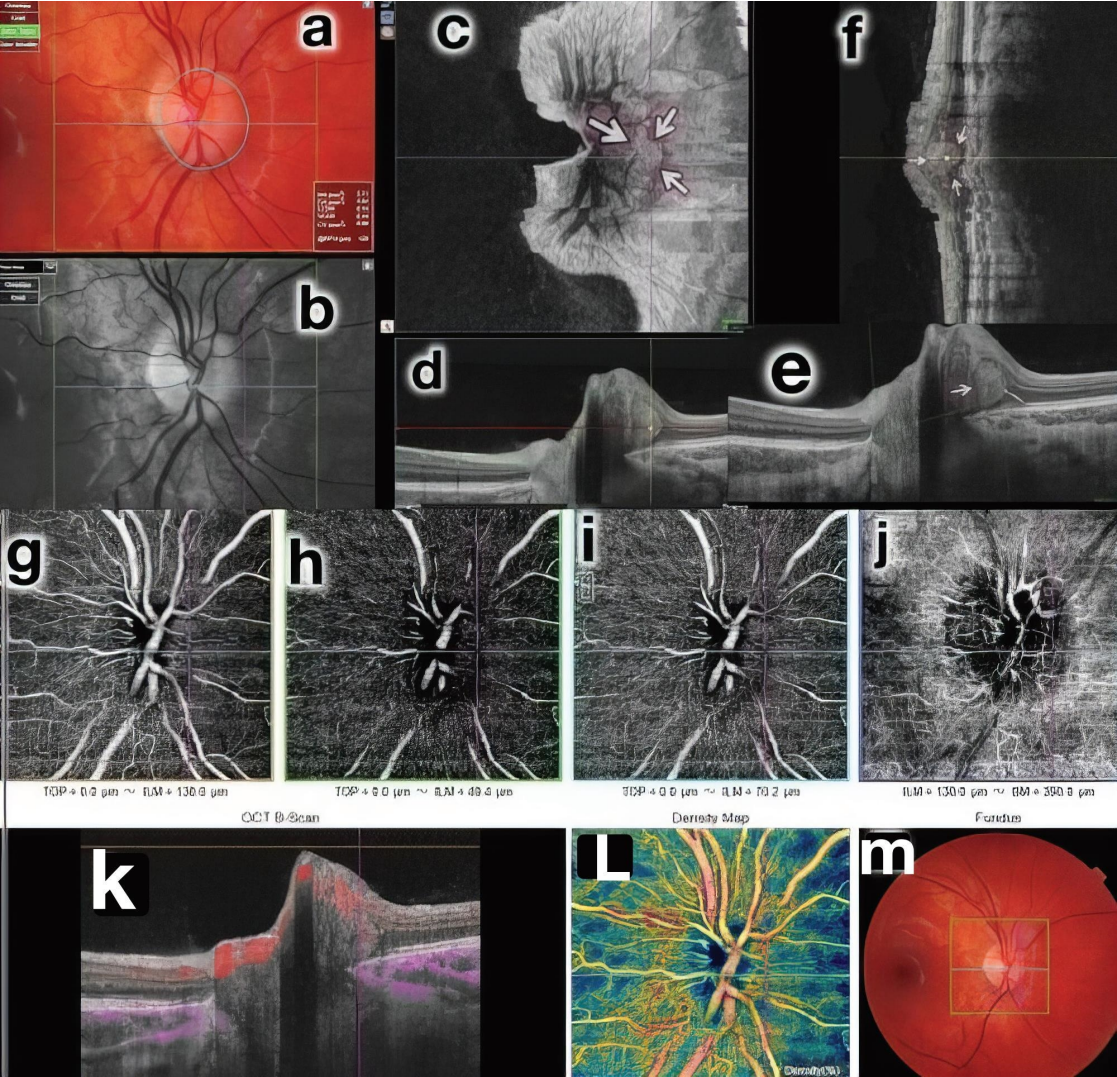
Multimodal study of the RE. Mild nasal elevation of the optic disc. Mild blurring of the nasal edge of the optic disc, normal macula (colour images [a, m], red free [b]); en face image: arrows delimit the PHOMS (c); SS‐OCT (d, e, f) locating the lesion; OCTA without changes in the anatomical conditions of capillary circulation in the different planes (g, h, i, j); focus of vascularization around PHOMS visible on B‐angio OCT (k) and homogeneous peri‐disc vascular density (l, m).

**Figure 2:**
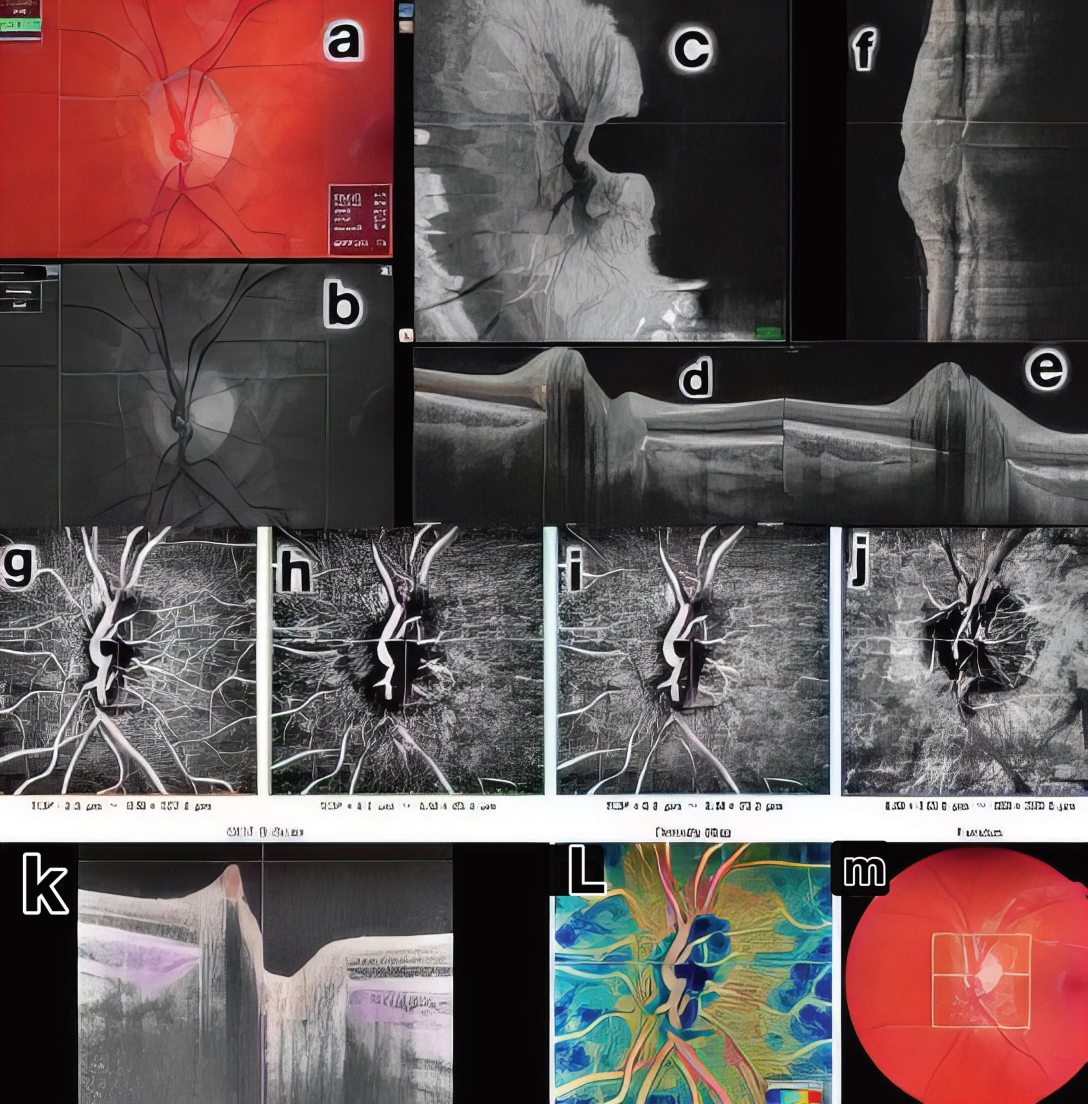
Multimodal study of the LE. Colour images (a, m), red‐free (b), en face OCT (c), OCT B (d, e, f), OCT A (g, h, i, j), B‐angio OCT (k) and vessel density (l, m): normal.

A 12‐year‐old Caucasian female patient was referred to our clinic for evaluation due to suspected sectoral edema in her right eye. Previous perimetry, intravenous fluorescein angiography, neuroimages and pentacam examinations were normal. The patient had a prior diagnosis of postural proprioceptive disorder and was using astigmatic correction (−1.00 180°) in both eyes. Prism correction was present, with a temporal base of 4 prismatic diopters (DP) and an upper base of 2 DP in the right eye, and a temporal base of 3 DP and an upper base of 2 DP in the left eye. Best‐corrected visual acuity was 20/20 in both eyes. Ocular motility examination revealed hyperfunction of the inferior oblique muscle (2/4) in both eyes, with a near point of convergence measured at 15 cm. Biomicroscopy findings were normal, and Ishihara colour plates showed no alterations. Fundoscopy revealed anomalous mild elevation of both optic discs without folds or subretinal fluid. Macular and retinal vessels appeared normal. OCT B‐scan and en face OCT demonstrated some fine tiny drusen and a well‐defined intradiscal hyperreflective body, without posterior shadow, leading to the diagnosis of PHOMS in the RE, with no evidence of fluid presence. OCT‐A did not reveal abnormal vascular abnormalities in vessel patterns, but intralesion vascularity was identified during angio B‐mode analysis. (Figures 1 and 2).

## 3. Discussion

PHOMS has been distinguished by the ODDS Consortium, indicating several characteristics that differentiate it from ODD. Firstly, unlike ODD, PHOMS presents as hyperreflective without a sharp outer margin or hyporeflective core. Additionally, PHOMS often surrounds large portions of the optic disc, mimicking funduscopically recognized pseudopapilledema. Unlike ODD, PHOMS does not exhibit autofluorescence and is not visible on B‐scan ultrasound despite its superficial location. Furthermore, this OCT finding has been observed in patients with papilledema without ODD, suggesting a possible correlation with the lateral bulging or herniation of distended axons into the peripapillary retina, as suggested by the histopathology of papilledema [1, 3]. Subsequent to these observations, ultrasound detection of PHOMS has been demonstrated [3, 9].

Since then, attempts to understand the pathophysiology of PHOMS have been made to understand the consequences of such findings in the prognosis and treatment of neuronal and optical diseases [9]. According to The Copenhagen Child Cohort 2000 Eye Study, PHOMS is the most common finding in pseudopapilledema in children [10]. Jorgensen et al. described that peripapillary retinal nerve fibre layer (RNFL) thickness decreases with age, as well as its volume and its ophthalmoscopic visibility [11]. A correlation between tobacco exposure during pregnancy and the presence of prelaminar hyperreflective lines was also found since prolonged toxin exposure during foetal organogenesis causes damage to the optic nerve. However, other studies have found PHOMS in patients who had no concomitant optic disease (as a casual finding, range 4%–19%) while also finding a high prevalence of PHOMS in patients with idiopathic intracranial hypertension (IIH). This finding could indicate a worse prognosis in NOIA patients and tends to disappear or regress as the edema subsides [7, 12, 13].

Histological differences in patients with PHOMS were also found, leading to the classification, by a recent review by Fraser et al., in three subtypes: (1) disc edema‐associated PHOMS, (2) ODD‐associated PHOMS and (3) anomalous disc‐associated PHOMS [4]. Therefore, based on the Copenhagen cohort study, it is necessary to investigate the presence or absence of pseudopapilledema in the fundus and imaging exams, to correlate with the appearance of PHOMS. As shown in the OCT Scans B and C, no fluids associated with the presence of intradiscal hyperreflexive bodies were observed, ruling out the possibility of a possible IIH, which could be causing a true papilledema. This finding is supported by a retrospective cohort study by Mezad‐Koursh et al., which was the first study that showed the prevalence of PHOMS in cases of pseudopapilledema in children [13]. However, although this article refers to a causal relationship between PHOMS and ODD, there is still no consensus on the pathophysiology of PHOMS, and it is still only a finding in imaging exams [14, 15].

More on the pathophysiology of PHOMS, not only do they accompany a plethora of ocular and neurological diseases, but also the true mechanisms behind such appearance, whether or not a worse prognosis can be correlated with them, are yet to be elucidated. A smaller scleral canal diameter and a trend towards a longer axial length have been associated with a myopic disc tilt as a primary cause of PHOMS [3]. Nevertheless, it is of extreme importance to determine the primary disease, as it will determine the course of treatment. Furthermore, by treating the baseline disease which led to axonal stasis, retinal ganglion cells and nerve fibre layer damage can be prevented.

Thus, the ODDS Consortium has since been used to elucidate differences between ODD and PHOMS. It has been pointed out that PHOMS are not detectable with other imaging modalities, unlike ODD, and its characteristic torus or partial torus 3D shape can be best seen with OCT [4, 16]. Subsequent to these observations, ultrasound, infrared photography, fluorescein angiography study detection of PHOMS has been demonstrated [3, 9, 17, 18]. Moreover, a study by Pichi et al. examined a paediatric cohort with tilted disc syndrome (TDS) both with SD‐OCT and with annual visual field testing, thus proving that 50% of the children presented “some amount of visual field defect at baseline, which completely regressed after maximal myopic correction” [18]. Another study found that only half of patients analyzed with ODD were found on ophthalmoscopy (56% of those with NA‐AION and 46% of those who were otherwise healthy), while also pointing out how ODD may have been underestimated in previous studies who did not possess of technology such as OCT [13–19].

OCT‐A has shown different patterns. Ahn et al. observed a capillary density substantially diminished in large but not in the medium or small sizes of PHOMS [20]. Xie et al., revealed blood flow signs in these hyperreflective lesions, that could indicate a similar capillary to the RNFL [17]. Borreli et al., showed a vascular complex that seems not to be in continuity with the physiological vasculature but may represent vessels deputed at the irrigation of the optic nerve [21]. Larger studies are needed to clarify these manifestations of PHOMS in multimodal imaging. PHOMS lesions appear less reflective than the surrounding drusen and do not produce posterior shadowing.

In general, the fundamental differences between PHOMS and ODD in OCT are as follows: reflectivity intensity, delineation of boundaries and posterior shadow formation. ODD are more reflective, have more easily delineated boundaries when superficial and tend to produce shadowing. Compressive effects on surrounding capillaries can be observed in OCT‐A, whereas PHOMS do not alter perfusional density.

In conclusion, PHOMS may serve as a biomarker for axoplasmic stasis, but its independent role as a risk factor for optical diseases and its consequences for nerve fibre function remains uncertain. Further longitudinal studies involving larger cohorts are needed to elucidate the pathogenesis of sectoral edema associated with PHOMS.

## Funding

No funding was received for this manuscript.

## Consent

Informed consent was obtained from the parents of the patient presented in the case report.

## Conflicts of Interest

The authors declare no conflicts of interest.

## Data Availability

The data that support the findings of this study are available from the corresponding author upon reasonable request
